# Facile One-Step Hydrothermal Synthesis of the rGO@Ni3V_2_O_8_ Interconnected Hollow Microspheres Composite for Lithium-Ion Batteries

**DOI:** 10.3390/nano10122389

**Published:** 2020-11-30

**Authors:** Faizan Ghani, In Wook Nah, Hyung-Seok Kim, JongChoo Lim, Afifa Marium, Muhammad Fazal Ijaz, Abu ul Hassan S. Rana

**Affiliations:** 1 Department of Chemical Engineering, Dongguk University, 30, Pildong-ro 1-gil, Jung-gu, Seoul 100-715, Korea; faizan@dgu.ac.kr; 2 Environment, Health, and Welfare Center, Korea Institute of Science and Technology, Hwarangno 14-gil 5, Seongbuk-gu, Seoul 02792, Korea; niw@kist.re.kr; 3 Center for Energy Storage Research, Korea Institute of Science and Technology, Hwarangno 14-gil 5, Seongbuk-gu, Seoul 02792, Korea; hskim0227@kist.re.kr; 4 Department of Intelligent Mechatronics Engineering, Sejong University, Seoul 05006, Korea; mariumafifa1440@gmail.com

**Keywords:** transition metal oxides, Ni_3_V_2_O_8_, reduced graphene oxide, hydrothermal synthesis, hollow microspheres, lithium ion battery

## Abstract

Low-cost, vanadium-based mixed metal oxides mostly have a layered crystal structure with excellent kinetics for lithium-ion batteries, providing high energy density. The existence of multiple oxidation states and the coordination chemistry of vanadium require cost-effective, robust techniques to synthesize the scaling up of their morphology and surface properties. Hydrothermal synthesis is one of the most suitable techniques to achieve pure phase and multiple morphologies under various conditions of temperature and pressure. We attained a simple one-step hydrothermal approach to synthesize the reduced graphene oxide coated Nickel Vanadate (rGO@Ni_3_V_2_O_8_) composite with interconnected hollow microspheres. The self-assembly route produced microspheres, which were interconnected under hydrothermal treatment. Cyclic performance determined the initial discharge/charge capacities of 1209.76/839.85 mAh g^−1^ at the current density of 200 mA g^−1^ with a columbic efficiency of 69.42%, which improved to 99.64% after 100 cycles. High electrochemical performance was observed due to high surface area, the porous nature of the interconnected hollow microspheres, and rGO induction. These properties increased the contact area between electrode and electrolyte, the active surface of the electrodes, and enhanced electrolyte penetration, which improved Li-ion diffusivity and electronic conductivity.

## 1. Introduction

Increasing demand for electronic appliances in daily life results in a quest for new energy materials for secondary batteries. In this era, the Lithium-ion battery (LIB) satisfies energy storage system requirements with low energy density [1]. Various materials, including carbonaceous, alloying, and transition metal oxides (TMOs), are studied as an anode of LIBs to improve energy and power densities [2,3]. Carbonaceous materials including graphite, carbon nanotubes, single-walled carbon nanotube (SWCNTs), and graphene oxides exhibiting intercalation chemistry are used as anodes of LIBs [4]. These materials are cost-effective and eco-friendly, but they deliver low energy density for LIBs. Graphite is commercially used as an anode material for LIBs and delivers almost 370 mAh g^−1^ of specific capacity. Conversely, alloying materials such as Si, Sn, Zn, etc., are abundant, eco-friendly, cost-effective, and provide high specific capacities. However, structural degradation because of extra volume expansion restricts their applicability. TMOs, such as Ni^II^O, Fe^III^_2_O_3_, Co^II/III^_3_O_4_, and Mn^II/III^_3_O_4_, are currently studied as replacements to carbonaceous materials with low energy density [5,6,7]. TMOs are low cost, environmentally friendly, and can deliver high energy density with optimal working potential. However, they still they have low electronic conductivity and Li^+^ ion diffusivity [6,8].

M_3_V_2_O_8_ (M = Ni^II^, Co^II/III^, Mn^II/III^, Zn^II^ and V^V/IV^) are new electroactive vanadate materials containing various oxidation states applied in energy storage systems like super capacitors, fuel cells, batteries, etc. These mixed TMOs have two distinct metal oxides, which can activate better redox reactions with superior specific capacities [9]. It is very hard to synthesize these kinds of mixed metal oxides because of their multiple oxidation states, anion role (O_2_^−^), and co-ordination chemistry. Also, it is challenging to control the related synthesis conditions of temperature, pressure, and time. There are very few reports on the synthesis of these materials and their applications [10,11,12,13,14]. Electronic conductivity and ionic transportation are the two main parameters that address the electrochemical performance of battery materials. These parameters can be enhanced through controlling the particle size, shape, phase, and porosity. To achieve these peculiar properties is a very challenging task and can be attained through different synthesis techniques [15,16]. These specific properties are obtained through various synthesis routes, such as co-precipitation, spray pyrolysis technique, and hydrothermal methods [10,17,18]. The hydrothermal process is already quite notorious for facile growth of different nanomaterials [19,20]. Hollow microspheres, rods, or tubular shape particles can modify surface properties and speculate porous nature to promote electrical and ionic conductivities. Ni_3_V_2_O_8_, comprised of Nickel oxide and a complex Vanadate structure, provides high specific capacities and is an appropriate choice of anode material for LIBs. However, fast structural degradation and sluggish diffusion kinetics hinder its practical application in energy storage systems [21,22]. Thus, maintaining structural integrity during the cycling process for high-capacity retention and columbic efficiency is a big challenge. As we know, coating carbonaceous materials or constructing composites can facilitate electronic conductivity and withstand volume changes during the charge/discharge of LIBs [23,24,25,26]. Reduced graphene oxide has a highly disordered surface, high specific surface area and better electronic conductivity, making it a suitable choice for constructing composites with Ni_3_V_2_O_8_ [24].

The objective of this work is to report a simple, facile one-step hydrothermal synthesis of the reduced graphene oxide interconnected hollow microspheres composite with Ni_3_V_2_O_8_. We synthesized a rGO@Ni_3_V_2_O_8_ hollow microspheres composite without the addition of any surfactant. This facile approach can enhance the specific surface area and porosity of the rGO@Ni_3_V_2_O_8_ microspheres composite, which effectively supports ionic transportation. The electrochemical performance of rGO@Ni_3_V_2_O_8_ was analyzed as anode material for LIBs. Discussions about electrochemical performance and rate capabilities are underlying.

## 2. Materials and Methods

All the materials were in analytical grades and used as received. Ni^II^ (CH_3_COO)_2_·4H_2_O was purchased from Kanto Chemical Industries, Tokyo, Japan. NH_4_V^V^O_3_ was purchased from Sigma-Aldrich (Saint Louis, MO, USA). Ethylene Glycol was purchased from Sigma-Aldrich, Daejung Chemical Industries, Busan, South Korea. The stoichiometric amounts of NH_4_V^V^O_3_ and Ni^II^ (CH_3_COO)_2_·4H_2_O were mixed with 10 mL of H_2_O each, separately, before being mixed together. After 1 h of mixing, 2 mL of ethylene glycol was added to the solution and stirred at room temperature for a further 4 h. The reduced graphene oxide was mixed into the precursor solution with rGO to a total cations ratio of 0.5:1 by weight, which was then stirred and sonicated for an hour. The solution was heated at 180 °C for 24 h in a 50 mL Teflon lined stainless steel autoclave (KISTEC, KIST, Seoul, Korea). Precipitates were collected, centrifuged, and washed several times with water and ethanol, respectively. They were then vacuum dried at 80 °C for 8 h to get the final rGO@Ni_3_V_2_O_8_ interconnected hollow microspheres composite. For comparison, the Ni_3_V_2_O_8_ interconnected hollow microspheres were also prepared through the same procedure without the addition of reduced graphene oxide.

The crystal phase, morphology, and topographical characteristics were measured using powder X-ray diffraction analysis (XRD; Rigaku D/MAX–2500 V/PC, Tokyo, Japan), scanning electron microscopy (FE–SEM; S–4200, Hitachi, High Technologies, Tokyo, Japan), and energy-filtered transmission electron microscopy (EF–TEM, Titan FEI, Corp., Hillsboro, OR, USA). N_2_ adsorption/desorption isotherm was studied via the BJH N_2_ adsorption instrument (Belsorp II mini BEL, Tokyo, Japan Inc.). The percentage weight loss of the rGO@Ni_3_V_2_O_8_ interconnected hollow microspheres composite was investigated through Thermogravimetric (TGA) analysis (TGA, Q-50, TA instruments Inc., New Castle, PA, USA) at 10 °C min^−1^ under an N_2_ atmosphere. Raman Spectroscopy (Renishaw, Seoul, Korea, Model at 532 nm) analysis was performed to study the molecular structure of the rGO@Ni_3_V_2_O_8_ interconnected hollow microspheres composite. The working electrode was prepared by mixing 70% rGO@Ni_3_V_2_O_8_, 20% Super P carbon additive, and 10% of 5% wt. poly (vinyl difluoride) binder in *N*–methyl pyrrolidone (NMP) solvent. The mass loading amount used around 0.8–1 mg cm^−3^ with 24 µm electrode thickness. The coin size (CR2032) electrodes were assembled in 1 M LiPF_6_ in an EC/DMC (1:1, *v*/*v*) electrolyte using polypropylene separators in an Ar filled glove box (Glove Box System (KK–011–AS), Korea KIYON, Seoul, Korea) with Li as a counter electrode. Electrochemical performance was studied via a galvanostatic/potentiostat system (Multi–Cycling Battery & Capacitor Test System, Series 4000, Thermo–Tech Co., Ltd., Seoul, Korea) in a voltage range of 3.0 V~0.01 V at a current density of 200 mA g^−1^. Cyclic Voltammometery (CV) and Electrochemical impedance spectroscopy (EIS) analysis were performed using a Biologic potentiostat/galvanostat Model VMP3 (BioLab, Inc. Pariset, France) at a scan rate of 0.1 mV s^−1^ and within the frequency range of 10 mHz−100 KHz.

## 3. Results and Discussion

**Figure 1:**
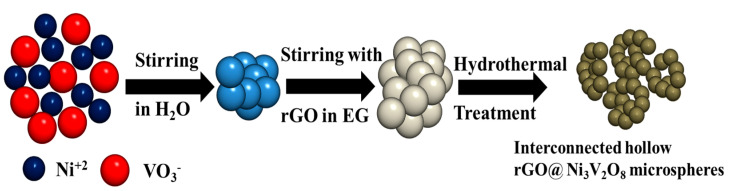
Systematic illustration mechanism of the rGO@Ni_3_V_2_O_8_ interconnected hollow microspheres composite.

**Figure 2:**
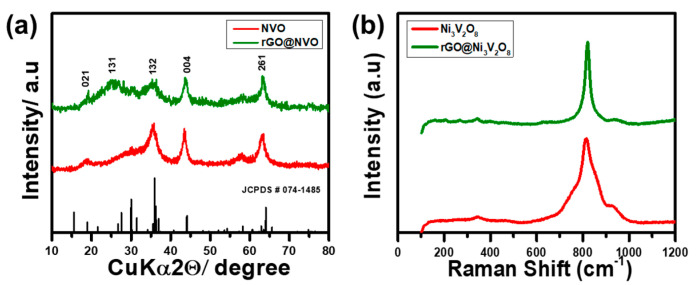
(**a**) X-ray diffraction analysis (XRD) patterns, and (**b**) Raman Spectroscopy Analysis of the Ni_3_V_2_O_8_ and rGO@Ni_3_V_2_O_8_ interconnected hollow microspheres composites.

Figure 1 shows the synthesis schematic of the rGO@Ni_3_V_2_O_8_ interconnected hollow microspheres composite. Initially, metal ions Ni^II^ and V^V^O_3_^−^ were produced when hydrolyzed for a certain time period. These ions, acting as electron donor and acceptor, chelated into one another to form a compact ligand. The insertion of ethylene glycol reduced the compact ligands and started nucleating nickel vanadate with a spherical shape. When reduced graphene oxide was added into the solution mixture, these microspheres entangled with one another on account of hydrothermal treatment for a specific time period. This entanglement generated interconnected hollow microspheres with a porous structure. The XRD patterns of the Ni_3_V_2_O_8_ and rGO@Ni_3_V_2_O_8_ composites are shown in Figure 2a, which shows that all the diffraction peaks match well with the orthorhombic crystal phase of JCPDS card # 074-1485. The broad peak around 25.34° is because of the incorporation of rGO into Ni_3_V_2_O_8_, which is confirmed in Figure S1. Rietveld refinement XRD analysis was performed using FullProf2000 software separately to the Ni_3_V_2_O_8_ microspheres and rGO@Ni_3_V_2_O_8_ interconnected hollow microspheres composites, and refined XRD results are shown in the Figure S4. Rietveld refinement analysis shows that the lattice parameters vary slightly from the standard unit cell data of Ni_3_V_2_O_8_. The increased unit cell volume for the rGO@Ni_3_V_2_O_8_ interconnected hollow microspheres is due to the presence of reduced graphene oxide. The crystallite sizes (calculated using the Scherrer equation), lattice parameters, and crystal volumes of the Ni_3_V_2_O_8_ and rGO@Ni_3_V_2_O_8_ composites are shown in Table 1.

**Figure 3:**
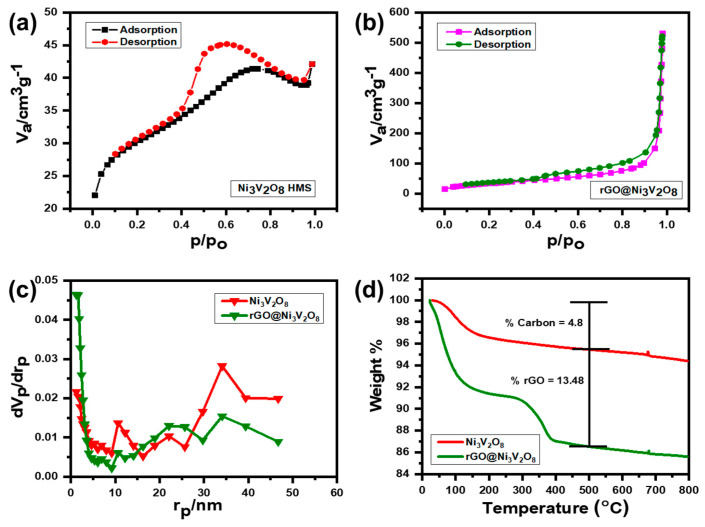
N_2_ adsorption/desorption isotherm of (**a**) Ni_3_V_2_O_8_ microspheres, (**b**) the rGO@Ni_3_V_2_O_8_ composite, (**c**) pore size distribution curves of the Ni_3_V_2_O_8_ microspheres and rGO@Ni_3_V_2_O_8_ microspheres composite, and (**d**) thermogravimetric (TGA) analysis of Ni_3_V_2_O_8_ microspheres and the rGO@Ni_3_V_2_O_8_ microspheres composite.

Raman spectroscopy is a highly sensitive technique for studying amorphous phases and molecular infringements. Figure 2b shows the Raman spectrum of the Ni_3_V_2_O_8_ and rGO@Ni_3_V_2_O_8_ composite with two obvious peaks around 380 cm^−1^ and 800 cm^−1^. These peaks correspond to the bending vibration mode of V-O-V and symmetric stretching mode of V-O for Ni_3_V_2_O_8_. The existence of these two peaks confirms the formation of Ni_3_V_2_O_8_ microspheres and a rGO@Ni_3_V_2_O_8_ composite. Furthermore, the Raman spectrum of the Ni_3_V_2_O_8_ microspheres and rGO@Ni_3_V_2_O_8_ composite are shown in Figure S2, which indicates two clear peaks consistent with the D band and G band at 1420.89 cm^−1^, 1618.87 cm^−1^ and 1349.52 cm^−1^, 1582.76 cm^−1^, respectively. The D band describes the defects induced in the graphene structure, whereas the G band explains the graphitic nature of reduced graphene oxide [27]. Ethylene glycol not only chelated the metal ions (Ni^II^ and V^V^O_3_^−^) but also reduced the carbon source during hydrothermal treatment and corresponded to the appearance of D and G bands in the Ni_3_V_2_O_8_ microspheres [28,29]. The high intensity ratio (I_D_/I_G_ = 1.07) of the rGO@Ni_3_V_2_O_8_ composite compared with I_D_/I_G_ = 0.66 of the Ni_3_V_2_O_8_ microspheres confirmed that there were large amounts of defects and grain boundaries on the surface. The high degree of disorder in the rGO@Ni_3_V_2_O_8_ interconnected hollow microspheres composite promoted Li^+^ ion diffusion and acted as a buffer to volume changes that occurred during the charge/discharge process. The N_2_ adsorption/desorption isotherms of the Ni_3_V_2_O_8_ microspheres and rGO@Ni_3_V_2_O_8_ composite were measured at 77 K and shown in Figure 3. The N_2_ adsorption/desorption isotherm of the Ni_3_V_2_O_8_ microspheres shown in the Figure 3a indicates that type IV isotherm with monolayer-multilayer adsorption proceeds at low pressure following the capillary condensation process at high pressure. This isotherm explains that the capillary condensation process occurs in mesopores with a limiting uptake up to high P/P0 pressure. The N_2_ adsorption/desorption isotherm of the rGO@Ni_3_V_2_O_8_ composite, shown in Figure 3b, conversely depicts the type III isotherm. This means that weak van der Waal interactions start to accumulate the adsorbate at low pressure, which continues to adsorb the lateral layers because of their strong interactions, leading to an unrestricted multilayer formation process in the filling of mesopores. The pore size distribution curves of the Ni_3_V_2_O_8_ microspheres and rGO@Ni_3_V_2_O_8_ composite are shown in Figure 3c. The pore size of electrode materials is an important parameter to control the diffusion kinetics of the Li+ ion within the electrode material. Increased pore size decreases the diffusion transportation length and reduces ionic diffusion resistance, which results in enhanced Li^+^ ion diffusivity. High mass transports conductivity results in a high specific capacity and cycle stability. Therefore, pore size is an effective parameter to control the rate performance of battery materials.

The specific surface area, pore sizes, and pore volumes of the Ni_3_V_2_O_8_ microspheres and rGO@Ni_3_V_2_O_8_ composite are shown in Table 2. Thermogravimetric (TGA) analysis of the Ni_3_V_2_O_8_ microspheres and rGO@Ni_3_V_2_O_8_ composite was performed under air atmosphere to measure the carbon content of rGO@Ni_3_V_2_O_8_ interconnected hollow microspheres composite, and the results are shown in Figure 3d. The TGA curves indicated that the moisture content evaporated when the temperature was increased up to 200 °C and the burning of carbon occurred between 400–500 °C. The percentage weight loss of the Ni_3_V_2_O_8_ microspheres and rGO@Ni_3_V_2_O_8_ composite was estimated at 4.80% and 13.48%, respectively. The presence of a small amount of carbon in the Ni_3_V_2_O_8_ microspheres was due to the addition of ethylene glycol, which reduced the precursors to nickel vanadate with a spherical shape. The addition of reduced graphene oxide to the solution resulted in increased amounts of carbon existence in the rGO@Ni_3_V_2_O_8_ composite, as is evident in Figure 3d. Therefore, it was inferred that the rGO@Ni_3_V_2_O_8_ interconnected hollow microspheres composite was successfully synthesized by the facile one-step hydrothermal technique, which has a high specific surface area and a porous nature to enhance electrical and ionic conductivities.

**Figure 4:**
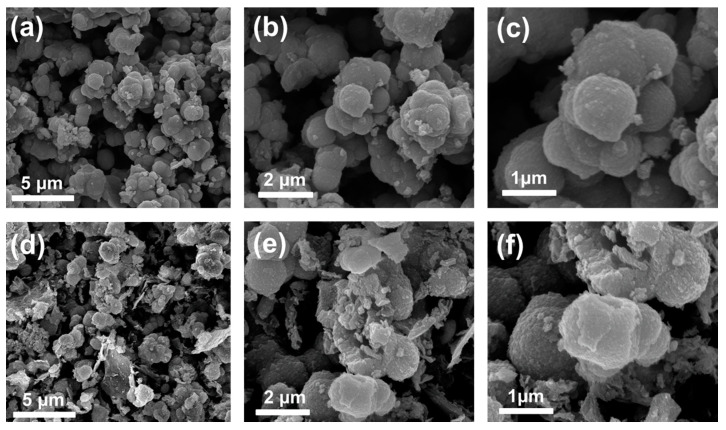
FESEM images of (**a**–**c**) Ni_3_V_2_O_8_ microspheres and (**d**–**f**) the rGO@Ni_3_V_2_O_8_ interconnected microspheres composite.

**Figure 5:**
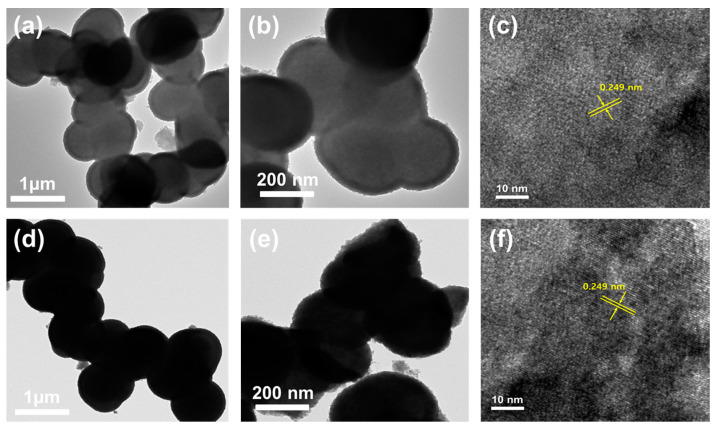
TEM and HRTEM images of (**a**–**c**) Ni_3_V_2_O_8_ microspheres and (**d**–**f**) rGO@Ni_3_V_2_O_8_ interconnected microspheres composite.

Figure 4 depicts the morphological characteristics of the Ni_3_V_2_O_8_ microspheres and rGO@Ni_3_V_2_O_8_ composite. Scanning electron microscopy (FESEM) images of the Ni_3_V_2_O_8_ microspheres, taken at various magnifications, are displayed in Figure 4a–c. The microspheres’ morphology shows that they were prominently interconnected with one another. The densification of the microspheres’ morphology is because of their amorphous nature. The microspheres’ sizes are estimated through ImageJ software and found to be 650 nm. Similarly, the SEM images of the rGO@Ni_3_V_2_O_8_ microspheres composite are shown in Figure 4d–f. The FESEM images depict the reduced graphene oxide sheets covered with microspheres. The presence of rGO and their amorphous nature result in their densification. The microspheres’ size was calculated as 675 nm. The energy dispersive spectroscopy (EDS) elemental mapping performed to investigate the existence of carbon in the Ni_3_V_2_O_8_ microspheres and rGO@Ni_3_V_2_O_8_ composite and its corresponding results are shown in Figures S5 and S6, respectively. The Figure S5 indicated a small amount of carbon presence in homogenously mixed Ni, V, and O elements. However, the EDS elemental mapping of the rGO@Ni_3_V_2_O_8_ composite showed that reduced graphene oxide was homogenously coated over the Ni_3_V_2_O_8_ microspheres. The amount of reduced graphene oxide was high when compared with the Ni_3_V_2_O_8_ microspheres. Besides, the topological features of the Ni_3_V_2_O_8_ microspheres and rGO@Ni_3_V_2_O_8_ composite were investigated through transmission electron microscopy (TEM) analyses and are shown in Figure 5. TEM images of Ni_3_V_2_O_8_ microspheres are shown in Figure 5a,b and confirm the formation of clusters of interconnected hollow microspheres.

The particle size calculated via TEM analyses was in accordance with the one calculated using FESEM analyses. The TEM images of the rGO@Ni_3_V_2_O_8_ microspheres composite are shown in Figure 5d,e and show rGO covering over the Ni_3_V_2_O_8_ interconnected hollow microspheres, confirming the rGO@Ni_3_V_2_O_8_ microspheres composite formation. The high resolution transmission electron microscopy (HRTEM) image of the Ni_3_V_2_O_8_ microspheres and rGO@Ni_3_V_2_O_8_ composites are shown in Figure 5c,f. The images explain the lattice fringe patterns with d spacing of 0.249 nm. The observed d spacing at the crystal plane of (122) agreed well with the JCPDS card # 074-1485 for the XRD pattern of Figure 2a. Hollow microspheres predominantly promoted Li^+^ ion diffusion and enhanced the penetration of electrolytes within the rGO@Ni_3_V_2_O_8_ microspheres composite, acting as a buffer against volume changes during the cycling process. Moreover, reduced graphene oxide facilitated the electron transfer from the composite to the current collector and also acted as a buffer against volume changes during the charge/discharge process.

**Figure 6:**
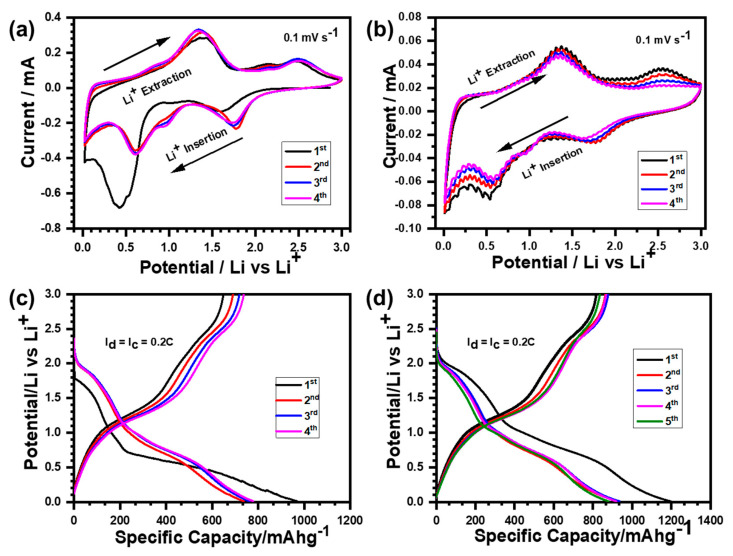
Cyclic Voltammogram (CV) curves at 0.1 mV s^−1^ of (**a**) the Ni_3_V_2_O_8_ microspheres, (**b**) the rGO@Ni_3_V_2_O_8_ composite, and galvanostatic discharge/charge profiles at 200 mA g^−1^ of (**c**) the Ni_3_V_2_O_8_ microspheres, and (**d**) the rGO@Ni_3_V_2_O_8_ composite, respectively.

The cyclic voltammetry (CV) of the Ni_3_V_2_O_8_ microspheres and rGO@Ni_3_V_2_O_8_ composite, investigated at 0.1 mV s^−1^ in a voltage range of 3.0 V~0.01 V for four cycles, is shown in Figure 6a,b. In the first cathodic sweep, lithiation started at around 2.0 V until the Ni_3_V_2_O_8_ microspheres were converted to NiO and Li_x+y_V_2_O_5_ at 1.55 V, with a change in the oxidation state of V^V^ to V^IV^. The solid electrolyte interface (SEI) layer started nucleating at around 0.8 V and shifted to 1.0 V in successive cycles, as shown in Figure 6a. A very clear reduction peak, observed at 0.45 V, was associated with the conversion of NiO to pure metallic Ni and Li_2_O, with some smaller reduction peaks linked to the lithiation of Li_x+y_V_2_O_5_. The cathodic peak at 0.45 V shifted to 0.55 V, and the conversion of the Ni_3_V_2_O_8_ microspheres to NiO and Li_x+y_V_2_O_5_ at 1.55 V moved to 1.78 V in successive cycles, with a change in the oxidation state of V^V^ to V^IV^. However, the broad peak observed at 1.3 V during the first anodic scan was for the pure metallic Ni oxidation to NiO, with a change in the V^II^ oxidation state to V^IV^. The second oxidation peak was found at 2.5 V and linked with the oxidation of Li_x+y_V_2_O_5_, with a change in the oxidation state of V^IV^ to V^V^. The overlapping of subsequent cycles indicated that the Ni_3_V_2_O_8_ microspheres had a similar electrochemical reaction and were suited to structural stability [24]. Consequently, the rGO@Ni_3_V_2_O_8_ composite reduced to NiO and Li_x+y_V_2_O_5_ at 1.78 V during the first cathodic scan, with a change in the oxidation state of V^V^ to V^IV^, as shown in Figure 6b. The solid electrolyte interface (SEI) layer was formed at around 0.9 V. The first cathodic scan indicated that there was a clear and wide peak at around 0.54 V, consistent with the conversion of NiO to pure metallic Ni and Li_2_O, which moved to 0.58 in successive cycles. While the broad oxidation peak observed at 1.3 V correspond to the oxidation of pure metallic Ni to NiO during the anodic scan, the peak observed at 2.5 V was associated with Li_x+y_V_2_O_5_ oxidation in the V^V^ oxidation state. Successive CV scans overlapped, demonstrating that the rGO@Ni_3_V_2_O_8_ composite followed a similar electrochemical reaction mechanism to the Ni_3_V_2_O_8_ microspheres. The presence of Li_x+y_V_2_O_5_ acted as a buffer for NiO to retain the structural integrity of Ni_3_V_2_O_8_ during the cycling process. Moreover, reduced graphene oxide also buffered the volume changes during the charging process, hence facilitating the structural integrity of the rGO@Ni_3_V_2_O_8_ composite. The discharge/charge voltage profiles of the Ni_3_V_2_O_8_ microspheres and rGO@Ni_3_V_2_O_8_ composite were investigated at 200 mA g^−1^ within the voltage window of 3.0 V~0.01 V and presented in Figure 6c,d. The first discharge curve shows that lithiation starts around 2.0 V with two distinct slopes at 1.5 V and 0.5 V, analogous to the reduction of the Ni_3_V_2_O_8_ microspheres to NiO and Li_x+y_V_2_O_5_ and then into pure metallic Ni and Li_2_O, as shown in Figure 6c. SEI layer formation was clearly observed at around 1.0 V. Similarly, two distinct charge slopes were observed at 1.3 V and 2.5 V, which were associated with the oxidation of pure metallic Ni into NiO and Li_x+y_V_2_O_5_.

The discharge/charge profiles of the following cycles indicated the same voltage plateaus as observed in the first cycle. These discharge/charge plateaus agreed well with the reduction/oxidation peaks of CV analysis, confirming a similar electrochemical mechanism throughout the cycling process. Likewise, the discharge/charge profile of the rGO@Ni_3_V_2_O_8_ composite was investigated at the current density of 200 mA g^−1^ within the voltage window of 3.0 V~0.01 V, as shown in Figure 6d. The first discharge curve indicated that lithiation was initiated at around 2.0 V, with two obvious slopes at 1.78 V and 0.55 V analogous to the conversion of the rGO@Ni_3_V_2_O_8_ composite to NiO, Li_x+y_V_2_O_5_ and then NiO into pure metallic Ni and Li_2_O. The formation of a solid electrolyte SEI layer was confirmed at around 1.0 V. The charging profile exhibited two discrete plateau formations at 1.3 V and 2.5 V, which correspond to the oxidation of pure metallic Ni to NiO and Li_x+y_V_2_O_5_. The subsequent cycles displayed similar voltage plateaus during the charge/discharge process, exhibiting the same electrochemical reaction chemistry. Furthermore, the voltage plateaus of the Ni_3_V_2_O_8_ microspheres and rGO@Ni_3_V_2_O_8_ composite shown in Figure 6c,d matched with the reduction/oxidation peaks of the respective CV analysis of Figure 6a,b. The discharge/charge plateaus matched with the reduction/oxidation peaks of the CV analysis, confirming the presence of reversible electrochemical reaction chemistry during the discharge/charge process.

**Figure 7:**
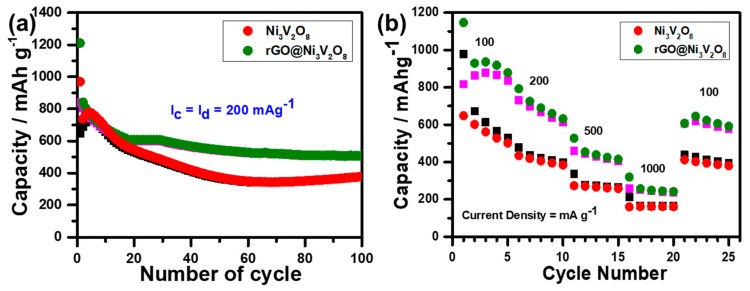
(**a**) Cyclic performance at the current density of 200 mA g^−1^ and (**b**) rate performance of the Ni_3_V_2_O_8_ microspheres and rGO@Ni_3_V_2_O_8_ interconnected hollow microspheres composite, respectively, (Red: Discharge, Black: Charge).

The cyclic performance of the Ni_3_V_2_O_8_ microspheres and rGO@Ni_3_V_2_O_8_ composite was investigated at the current density of 200 mA g^−1^, and the respective results are shown in Figure 7a. It is evident that the Ni_3_V_2_O_8_ microspheres and rGO@Ni_3_V_2_O_8_ composite deliver the initial discharge/charge capacities of 969.52/648.86 mAh g^−1^ and 1209.76/839.85 mAh g^−1^ with a columbic efficiency of 66.92% and 69.42%, respectively, which approaches 99% after 100 cycles. The stability of the columbic efficiency of the Ni_3_V_2_O_8_ microspheres and rGO@Ni_3_V_2_O_8_ composite up to 100 cycles is shown in Figure S3. The initial low columbic efficiency of the rGO@Ni_3_V_2_O_8_ composite is due to the presence of amorphous graphene oxide [29,30,31]. The capacity fading in the initial 15 cycles is associate with unstable SEI layer formation because of the complex lithiation chemistry of Li_x+y_V_2_O_5_. Cycle stability was induced because of rGO. The incorporation of rGO facilitated the electronic conductivity to the rGO@Ni_3_V_2_O_8_ composite and buffered the volume changes during the cycling process, thus retaining structural integrity. The rate capability of the Ni_3_V_2_O_8_ microspheres and rGO@Ni_3_V_2_O_8_ composite were measured at the current densities of 100, 200, 500, and 1000 mA g^−1^ for 5 cycles each and are shown in Figure 7b. The discharge capacities of the Ni_3_V_2_O_8_ microspheres were investigated at 978.09 mAh g^−1^, 478.10 mAh g^−1^, 336.15 mAh g^−1^, and 212.06 mAh g^−1^, respectively. However, the discharge capacities of the rGO@Ni_3_V_2_O_8_ composite were measured as 1147.24 mAh g^−1^, 792.63 mAh g^−1^, 527.49 mAh g^−1^, and 319.33 mAh g^−1^, respectively. Figure 7b demonstrates the specific discharge/charge capacities of 527.49/505.25 mAh g^−1^, even at a high current density of 500 mA g^−1^, which is much higher than that of commercial graphite anode material (370 mAh g^−1^). High rate performance and cycle life make the rGO@Ni_3_V_2_O_8_ interconnected hollow microspheres composite an appropriate choice of anode material for LIBs. The high rate and cyclic performance are connected with higher surface area and pore size, which enhance the contact area between electrode and electrolyte and allow more electrolytes to penetrate the rGO@Ni_3_V_2_O_8_ interconnected hollow microspheres composite, sufficiently improving its ionic conductivity. Furthermore, the inclusion of reduced graphene oxide in the rGO@Ni_3_V_2_O_8_ interconnected hollow microspheres composite imparts electronic conductivity and buffers the volume changes during the cycling process, which retains its structural integrity.

**Figure 8:**
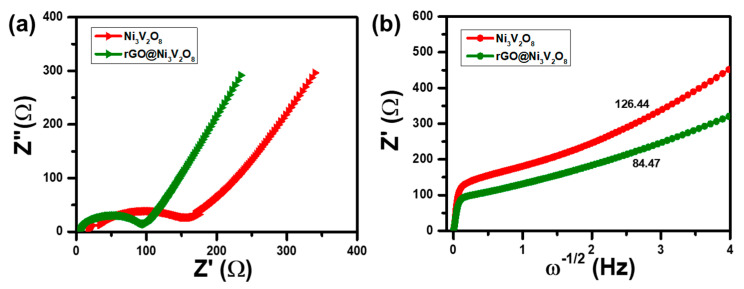
(**a**) Nyquist plot after 100th cycles and (**b**) Linear Warburg diffusion constant of the Ni_3_V_2_O_8_ microspheres and rGO@Ni_3_V_2_O_8_ interconnected hollow microspheres composite, respectively

In addition, the electrochemical reaction kinetic was investigated through impedance spectroscopy analysis, and results are shown in Figure 8. The Nyquist plot shown in Figure 8a indicates the internal solution resistance (R_s_) of the Ni_3_V_2_O_8_ microspheres and rGO@Ni_3_V_2_O_8_ composite as 17.35 Ω and 4.76 Ω after 100 cycles, respectively. The charge transfer resistance (R_ct_) corresponds to the semi-circle in the medium frequency range of the Ni_3_V_2_O_8_ microspheres and the rGO@Ni_3_V_2_O_8_ interconnected hollow microspheres composite and is measured as 175 Ω and 100 Ω, respectively. The charge transfer resistance demonstrated that electronic conductivity increased in the case of the rGO@Ni_3_V_2_O_8_ interconnected hollow microspheres composite because of the existence of reduced graphene oxide. Furthermore, the Warburg impedance was consistent with the sloping line in the low frequency region and confirmed Li^+^ ion diffusion.

The letter σ is the slope of linear fit of Z_real_ vs. ω^−1/2^, which is evident in Figure 8b and is consistent with the Warburg impedance. This indicates that the rGO@Ni_3_V_2_O_8_ interconnected hollow microspheres composite exhibits higher diffusivity due to rGO incorporation, which shortens the Li^+^ diffusion length.

## 4. Conclusions

A facile one-step hydrothermal technique synthesized rGO@Ni_3_V_2_O_8_ interconnected hollow microspheres composites. Microspheres were characterized with numerous analyses techniques, including XRD, BET, and TEM, which revealed their porous nature with high surface area, crystalline phase, and rGO@Ni_3_V_2_O_8_ composite formation. The high surface area and porous nature improved and facilitated Li^+^ ion diffusion, whereas the presence of rGO promoted electronic conductivity and buffered the volume changes during the charging process. Increased Li^+^ ion diffusivity and conductivity enhanced the electrochemical performance of the rGO@Ni_3_V_2_O_8_ interconnected hollow microspheres composite.

## Figures and Tables

**Table 1:** Summary of the lattice parameters, crystal volume and crystallite sizes of the Ni_3_V_2_O_8_ microspheres and rGO@Ni_3_V_2_O_8_ interconnected hollow microspheres composites, respectively.

Samples	Parameters			Volume V = a × b × c	Crystallite Size (nm)
	a	b	c		
Standard	8.240	11.380	5.906	553.812	-
Ni_3_V_2_O_8_	8.239	11.380	5.905	553.651	5.09
rGO@Ni_3_V_2_O_8_	8.207	11.415	5.975	559.755	116.07

**Table 2:** Summary of the physicochemical properties of the Ni_3_V_2_O_8_ microspheres and rGO@Ni_3_V_2_O_8_ interconnected hollow microspheres composite, respectively.

Samples	Pore Volume (m^3^ g^−1^)	Pore Size (nm)	BET Area (m^3^ g^−1^)	Carbon % wt.	I_D_/I_G_
Ni_3_V_2_O_8_	5.292 × 10^−8^	2.627	99.193	4.80	0.66
rGO@Ni_3_V_2_O_8_	8.221 × 10^−7^	30.944	106.270	13.48	1.07

## Supplementary Materials

The following are available online at https://www.mdpi.com/2079-4991/10/12/2389/s1, Figure S1: XRD analysis of as synthesis reduced graphene oxide, Figure S2: Raman spectroscopy analysis of Ni_3_V_2_O_8_ microspheres and rGO@Ni_3_V_2_O_8_ interconnected hollow microspheres composite, Figure S3 Columbic efficiency *vs* number of cycle graph of Ni_3_V_2_O_8_ microspheres and rGO@Ni_3_V_2_O_8_ interconnected hollow microspheres composite, Figure S4: Rietveld refinement of XRD analysis of as synthesis (a) Ni_3_V_2_O_8_ microspheres, and (b) rGO@Ni_3_V_2_O_8_ interconnected hollow microspheres composites, Figure S5: EDS Elemental analysis of as synthesis Ni_3_V_2_O_8_ microspheres, (a) C, (b) O, (c) Ni, and (d) V. Figure S6: EDS Elemental analysis of as synthesis rGO@Ni_3_V_2_O_8_ microspheres, (a) C, (b) O, (c) Ni, and (d) V.
