# Supplementary material for: Facile One-Step Hydrothermal Synthesis of the rGO@Ni3V2O8 Interconnected Hollow Microspheres Composite for Lithium-Ion Batteries
Source: Nanomaterials (Basel). 2020 Nov 30;10(12):2389. doi: 10.3390/nano10122389 (PMC7760731; doi:10.3390/nano10122389)
Supplement: Supplementary file 1 [file nanomaterials-10-02389-s001.pdf]

nanomaterials

Supplementary Materials

Facile One-Step Hydrothermal Synthesis of the rGO@Ni3V_2O_8 Interconnected Hollow Microspheres Composite for Lithium-Ion Batteries

Faizan Ghani [1,2], In Wook Nah [2], Hyung Seok Kim [3], JongChoo Lim [1,*], Afifa Marium [4], Muhammad Fazal Ijaz [4,*] and Abu ul Hassan S. Rana [4,*]

[1] Department of Chemical Engineering, Dongguk University, 30, Pildong-ro 1-gil, Jung-gu, Seoul 100-715, Korea; faizan@dgu.ac.kr
[2] Environment, Health, and Welfare Center, Korea Institute of Science and Technology, Hwarangno 14-gil 5, Seongbuk-gu, Seoul 02792, Korea; niw@kist.re.kr
[3] Energy Storgae Center, Korea Institute of Science and Technology, Hwarangno 14-gil 5, Seongbuk-gu, Seoul 02792, Korea; hskim0227@kist.re.kr
[4] Department of Intelligent Mechatronics Engineering, Sejong University, Seoul 05006, Korea; mariumafifa1440@gmail.com
* Correspondence: jongchoo@dongguk.edu (J.L.); fazal@sejong.ac.kr (M.F.I.); rana@sejong.ac.kr (A.u.H.S.R.)

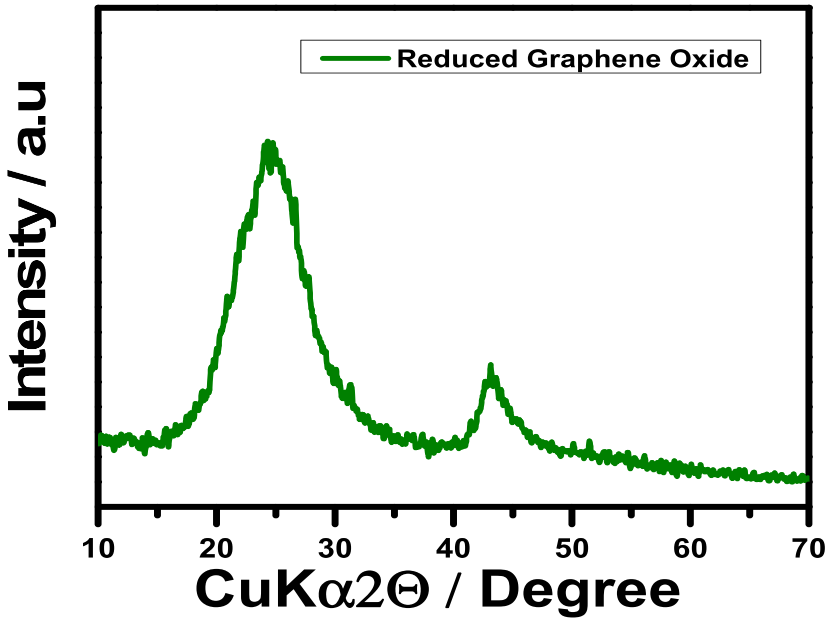

Figure S1. XRD analysis of as synthesis reduced graphene oxide.

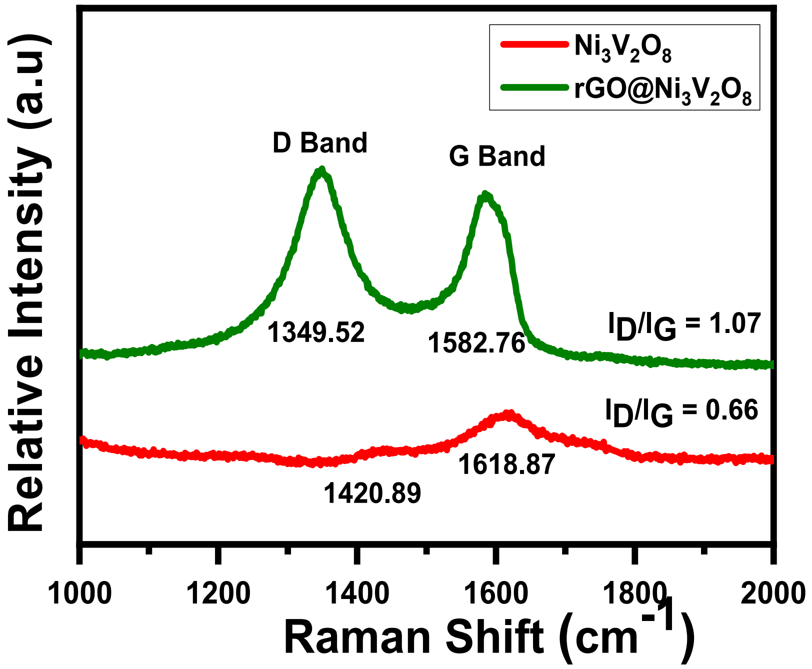

Figure S2. Raman spectroscopy analysis of $Ni_3V_2O_8$ microspheres and $rGO@Ni_3V_2O_8$ interconnected hollow microspheres composite.

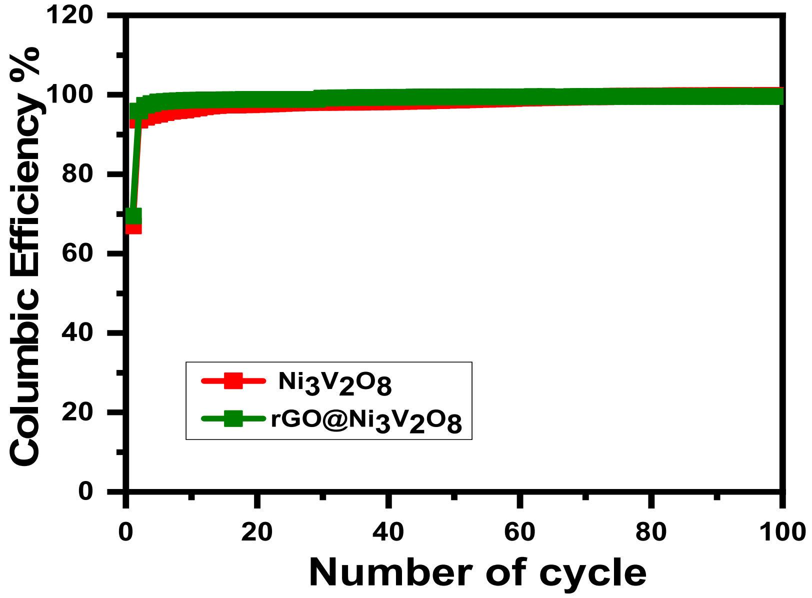

Figure S3. Columbic efficiency *vs* number of cycle graph of $Ni_3V_2O_8$ microspheres and $rGO@Ni_3V_2O_8$ interconnected hollow microspheres composite.

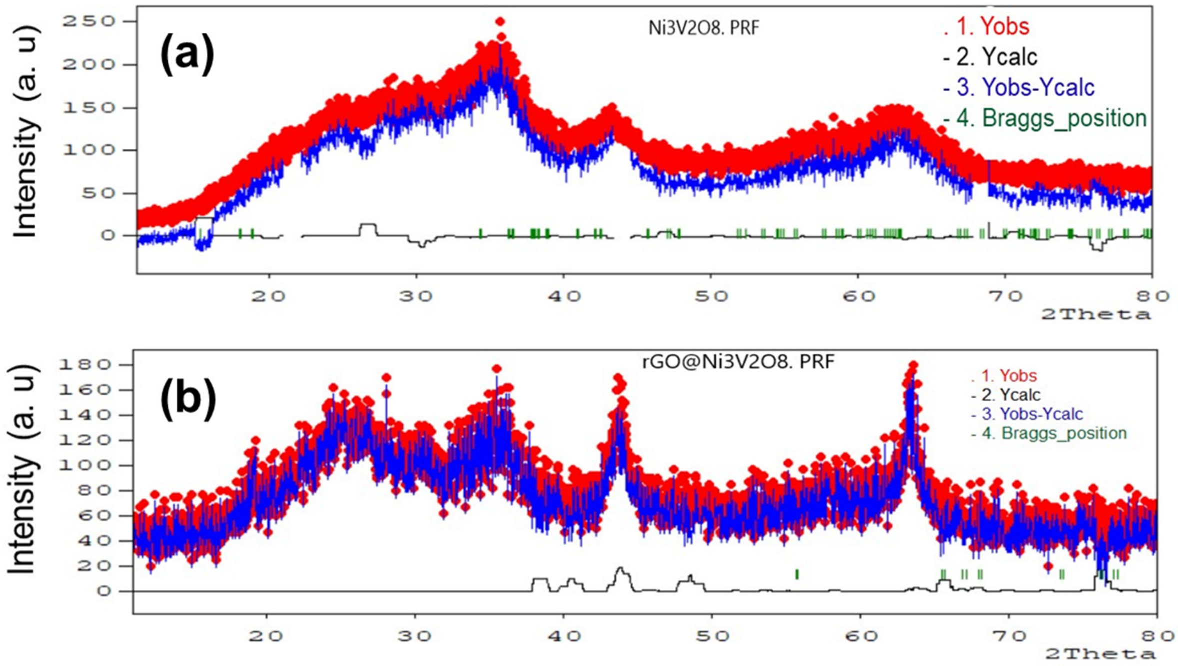

Figure S4. Rietveld refinement of XRD analysis of as synthesis (**a**) $Ni_3V_2O_8$ microspheres, and (**b**) rGO@$Ni_3V_2O_8$ interconnected hollow microspheres composites.

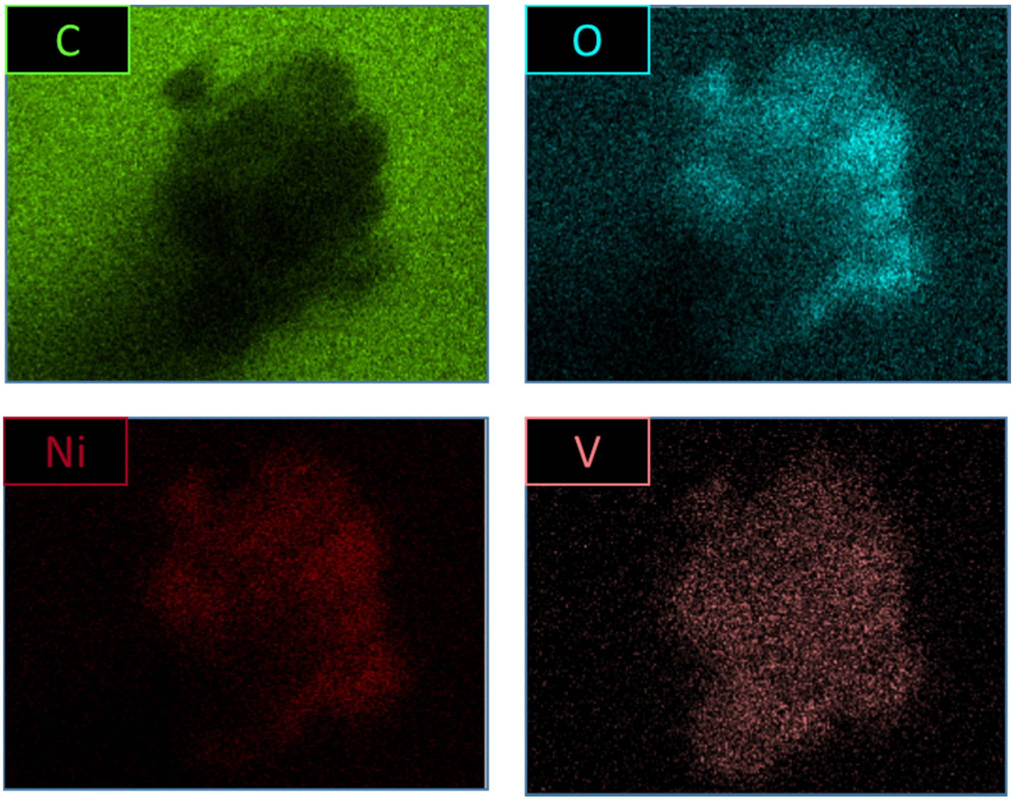

Figure S5. EDS Elemental analysis of as synthesis $Ni_3V_2O_8$ microspheres, (**a**) C, (**b**) O, (**c**) Ni, and (**d**) V.

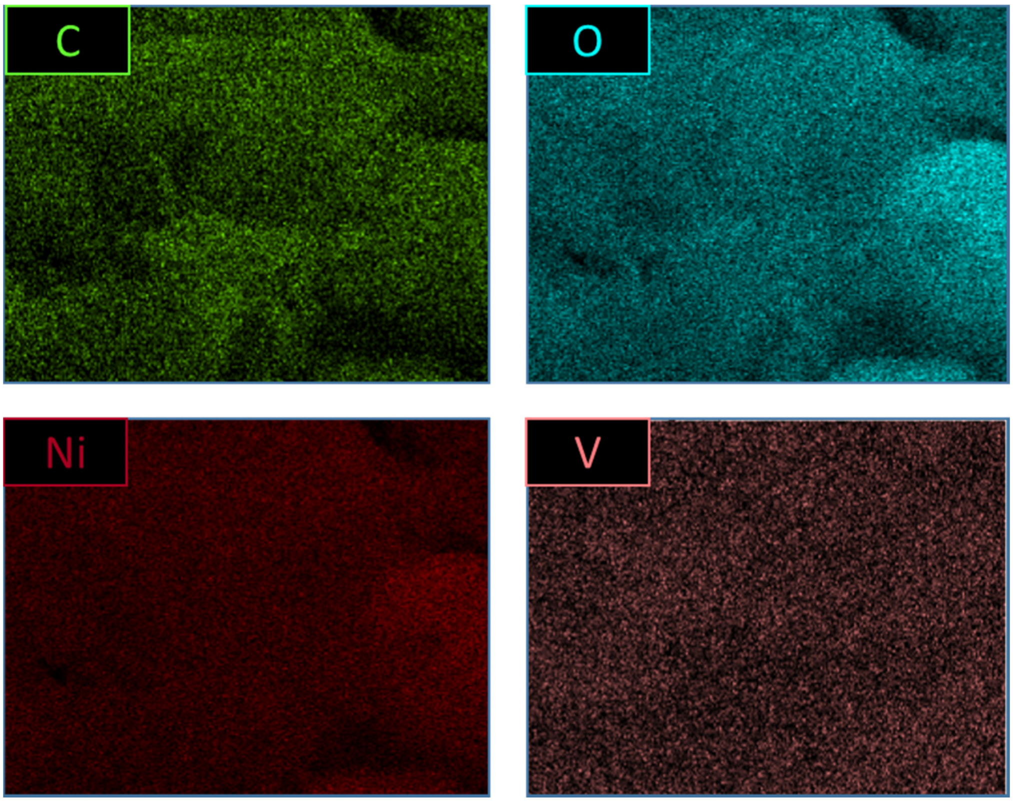

Figure S6. EDS Elemental analysis of as synthesis rGO@$Ni_3V_2O_8$ microspheres, (**a**) C, (**b**) O, (**c**) Ni, and (**d**) V.
